# A cost-utility analysis between decompression only and fusion surgery for elderly patients with lumbar spinal stenosis and sagittal imbalance

**DOI:** 10.1038/s41598-022-24784-4

**Published:** 2022-11-27

**Authors:** Young Il Won, Chi Heon Kim, Hee-Pyoung Park, Sun Gun Chung, Woon Tak Yuh, Shin Won Kwon, Seung Heon Yang, Chang-Hyun Lee, Yunhee Choi, Sung Bae Park, John M. Rhee, Kyoung-Tae Kim, Chun Kee Chung

**Affiliations:** 1 Department of Neurosurgery, Chungnam National University Sejong Hospital, 20, Bodeum 7-ro, Sejong, 30099 Republic of Korea; 2 Department of Neurosurgery, Seoul National University Hospital, 101, Daehak-ro, Jongno-gu, Seoul, 03080 Republic of Korea; 3 Department of Neurosurgery and Medical Device Development, Seoul National University College of Medicine, 103 Daehak-ro, Jongno-gu, Seoul, 03080 Republic of Korea; 4 Department of Anesthesiology and Pain Medicine, Seoul National University College of Medicine and Hospital, 101, Daehak-ro, Jongno-gu, Seoul, 03080 Republic of Korea; 5 Department of Rehabilitation Medicine, Seoul National University College of Medicine and Hospital, 103 Daehak-ro, Jongno-gu, Seoul, 03080 Republic of Korea; 6 Department of Neurosurgery, Hallym University Dongtan Sacred Heart Hospital, 7 Keunjaebong-gil, Hwaseong-si, Gyeonggi-do 18450 Republic of Korea; 7 Department of Neurosurgery, Incheon Veterans Hospital, 138, Inju-daero, Michuhol-gu, Incheon, 22182 Republic of Korea; 8 Department of Neurosurgery, Seoul National University Bundang Hospital, 82, Gumi-ro 173 Beon-gil, Bundang-gu, Seongnam-si, Gyeonggi-do 13620 Republic of Korea; 9 Division of Medical Statistics, Medical Research Collaborating Center, Seoul National University Hospital, 101, Daehak-ro, Jongno-gu, Seoul, 03080 Republic of Korea; 10 Department of Neurosurgery, Seoul National University Boramae Hospital, Boramae Medical Center 20, Boramae-ro 5-gil, Dongjak-gu, Seoul, 07061 Republic of Korea; 11 Department of Orthopaedic Surgery, Emory University School of Medicine, Atlanta, GA 30322 USA; 12 Department of Neurosurgery, Kyungpook National University Hospital, 130 Dongdeok-ro, Jung-gu, Daegu, 41944 Republic of Korea; 13 Department of Neurosurgery, School of Medicine, Kyungpook National University, 680 Gukchaebosang-ro, Jung-gu, Daegu, 41944 Republic of Korea; 14 Department of Brain and Cognitive Sciences, Seoul National University, 101, 1, Gwanak-ro, Gwanak-gu, Seoul, Republic of Korea; 15 Department of Neurosurgery, Seoul National University College of Medicine, Seoul National University Hospital, 103 Daehak-ro, Jongno-gu, Seoul, 03080 Republic of Korea

**Keywords:** Musculoskeletal system, Pain, Quality of life

## Abstract

Lumbar spinal stenosis (LSS) and sagittal imbalance are relatively common in elderly patients. Although the goals of surgery include both functional and radiological improvements, the criteria of correction may be too strict for elderly patients. If the main symptom of patients is not forward-stooping but neurogenic claudication or pain, lumbar decompression without adding fusion procedure may be a surgical option. We performed cost-utility analysis between lumbar decompression and lumbar fusion surgery for those patients. Elderly patients (age > 60 years) who underwent 1–2 levels lumbar fusion surgery (F-group, n = 31) or decompression surgery (D-group, n = 40) for LSS with sagittal imbalance (C7 sagittal vertical axis, C7-SVA > 40 mm) with follow-up ≥ 2 years were included. Clinical outcomes (Euro-Quality of Life-5 Dimensions, EQ-5D; Oswestry Disability Index, ODI; numerical rating score of pain on the back and leg, NRS-B and NRS-L) and radiological parameters (C7-SVA; lumbar lordosis, LL; the difference between pelvic incidence and lumbar lordosis, PI-LL; pelvic tilt, PT) were assessed. The quality-adjusted life year (QALY) and incremental cost-effective ratio (ICER) were calculated from a utility score of EQ-5D. Postoperatively, both groups attained clinical and radiological improvement in all parameters, but NRS-L was more improved in the F-group (*p* = 0.048). ICER of F-group over D-group was 49,833 US dollars/QALY. Cost-effective lumbar decompression may be a recommendable surgical option for certain elderly patients, despite less improvement of leg pain than with fusion surgery.

## Introduction

Recently, many countries have been entering aging or aged societies^1^. Lumbar spinal stenosis (LSS) is the most common lumbar degenerative disease and incurs a large socioeconomic burden on those societies^2,3^. Since LSS can occur along with other conditions, such as sagittal imbalance, decision-making and treatment options may vary widely^4–6^. For medically intractable patients with LSS and sagittal imbalance, lumbar decompression surgery with or without instrumented fusion may be a surgical option if the main symptom of a patient is neurogenic intermittent claudication or pain, and forward-stooped posture is not a major problem for the patient^5,7,8^. The goals of surgery include both improvement of functional status and sagittal balance, but achieving both goals may not be necessary for all patients. Although it is known that sagittal imbalance can be associated with poor functional outcomes, the criteria for imbalance are based on normal healthy adults and may not universally apply to elderly patients, especially those in Asian societies who prefer to sit on the floor^9,10^. Moreover, instrumented fusion surgery is associated with a higher cost and complication rate than decompression surgery^3,11,12^. A randomized controlled trial study showed that the clinical outcome of decompression surgery was comparable to that of fusion surgery for patients with stable LSS^12,13^. Recent studies showed that sagittal balance was improved after lumbar decompression surgery without instrumented fusion for LSS, although the improvement of sagittal balance may be less than that of fusion surgery^4,5,14,15^. Symptomatic improvement without complete radiological correction may be a suggestable option for elderly patients, considering the cost and morbidity of fusion surgery^5,15,16^. However, a residual sagittal imbalance may influence the clinical outcome and medical cost^5,17^. In this regard, we analyzed cost-utility analysis between lumbar decompression surgery and lumbar fusion surgery for patients with LSS and sagittal imbalance.

## Methods

We retrospectively reviewed a prospectively collected database to identify elderly patients who underwent 1–2 levels lumbar decompression surgery (D-group) or fusion surgery (F-group) for LSS with sagittal imbalance from 2014 to 2018. The included patients were more than 60 years^18^ old and had severe central LSS, medically intractable pain or claudication for more than 3 months, C7 sagittal vertical axis (C7-SVA) > 40 mm, and postoperative follow-up for at least 2 years. Patients with instability on dynamic radiographs (motion of > 3 mm at the level of listhesis, as measured on flexion–extension radiographs of the lumbar spine)^6,12^, grade 2 or higher spondylolisthesis, degenerative scoliosis more than 20 degrees^6,12^, three or more segment surgeries, prior lumbar surgery, or neurological disease (such as Parkinson’s disease) were excluded from this study. All cases were operated on by spine surgeons with more than ten years of experience. A posterior unilateral approach and bilateral decompression or oblique lumbar interbody fusion (OLIF) are routine surgical methods for patients with LSS. As a routine practice, the pros and cons of decompression only and fusion surgery were explained in a shared decision-making process with patients preoperatively. Perioperative risk assessed by ASA classification and osteoporosis evaluated by bone mineral density (BMD) measurement were considered, and both patients and surgeons favored decompression surgery.

The surgical decision and goal were reaffirmed with the patient one day before the operation. This study was conducted in accordance with the Declaration of Helsinki and the Guideline for Good Clinical Practice. The study protocol was approved by the Seoul National University Hospital ethics committee/institutional review board (IRB No.: H-2101-080-1187). The Seoul National University Hospital ethics committee/institutional review board approved the exemption of patient informed consent due to the retrospective nature of this study.

### Surgical techniques and hospital course

Posterior lumbar decompression was performed in the prone position using an approximately 3 cm midline skin incision. A tubular retractor was placed on the lamina of the symptomatic side, and unilateral laminotomy was performed using a rotary drill. Then, the ligamentum flavum (LF) was removed from the bilateral lamina by using a curved curette and a rongeur. For OLIF, the patient was positioned in the right lateral decubitus. The external and internal oblique and transverse muscle fibers were dissociated after a 5–6 cm skin incision. The peritoneum and retroperitoneal fat were swept anteriorly to expose the belly of the psoas muscle. Retroperitoneal structures were gently mobilized and protected by retractor blades to expose the corridor between the psoas muscle and the aorta. Then, the disc space was prepared for fusion, and the size of the interbody cage was determined using trial cages. A polyetheretherketone cage filled with cancellous allograft bone chips mixed with autologous bone marrow was implanted under fluoroscopic guidance. For L2-5, cages had 6 of lordosis, and the height was determined considering adjacent segments and desired lumbar lordosis, with heights ranging from 10, 12, to 14 mm. The width of the cage was selected to reach the bilateral margins of the vertebral body, ranging from 40, 45, to 50 mm in length. For L5-S1, the size and angle of the cages were combinations of 8 or 12° lordosis and 10, 12, 14 or 16 mm in height. After closure of the surgical wound at the flank, patients were flipped to the prone position, and bilateral percutaneous pedicle screw fixation was performed.

All patients in both groups were encouraged to ambulate from the day of surgery and were discharged 3–4 days later. Postoperative magnetic resonance imaging (MRI) was taken from all patients to confirm decompression. A lumbar brace was recommended for 1–3 months for patients with fusion surgery and ctivities of daily living allowed after discharge. Patients were allowed to return to office work within a month as tolerated. Strenuous exercise or hard work was allowed 3 months after surgery if there was no issue of mechanical failure.

### Clinical assessment

Clinical outcomes were assessed using the Euro-Quality of Life-5 Dimension (EQ-5D), the Korean version of the Oswestry Disability Index (ODI)^19^, and the numerical rating score of pain on the back (NRS-B) and leg (NRS-L). The EQ-5D is a standardized instrument for measuring generic health status^20^. It consists of five health dimensions: mobility, self-care, usual activities, pain/discomfort, and anxiety/depression. The responses to the EQ-5D descriptive system can be converted into a single number called an index value, which ranges from ‘death’ (0 point) to ‘perfect health’ (1 point). These numbers serve as quality adjustment weights^20^. All patients were asked to complete questionnaires that included those parameters before the operation and 3, 6, and 12 months postoperatively and yearly thereafter in the outpatient clinic.

### Radiological assessment

**Figure 1:**
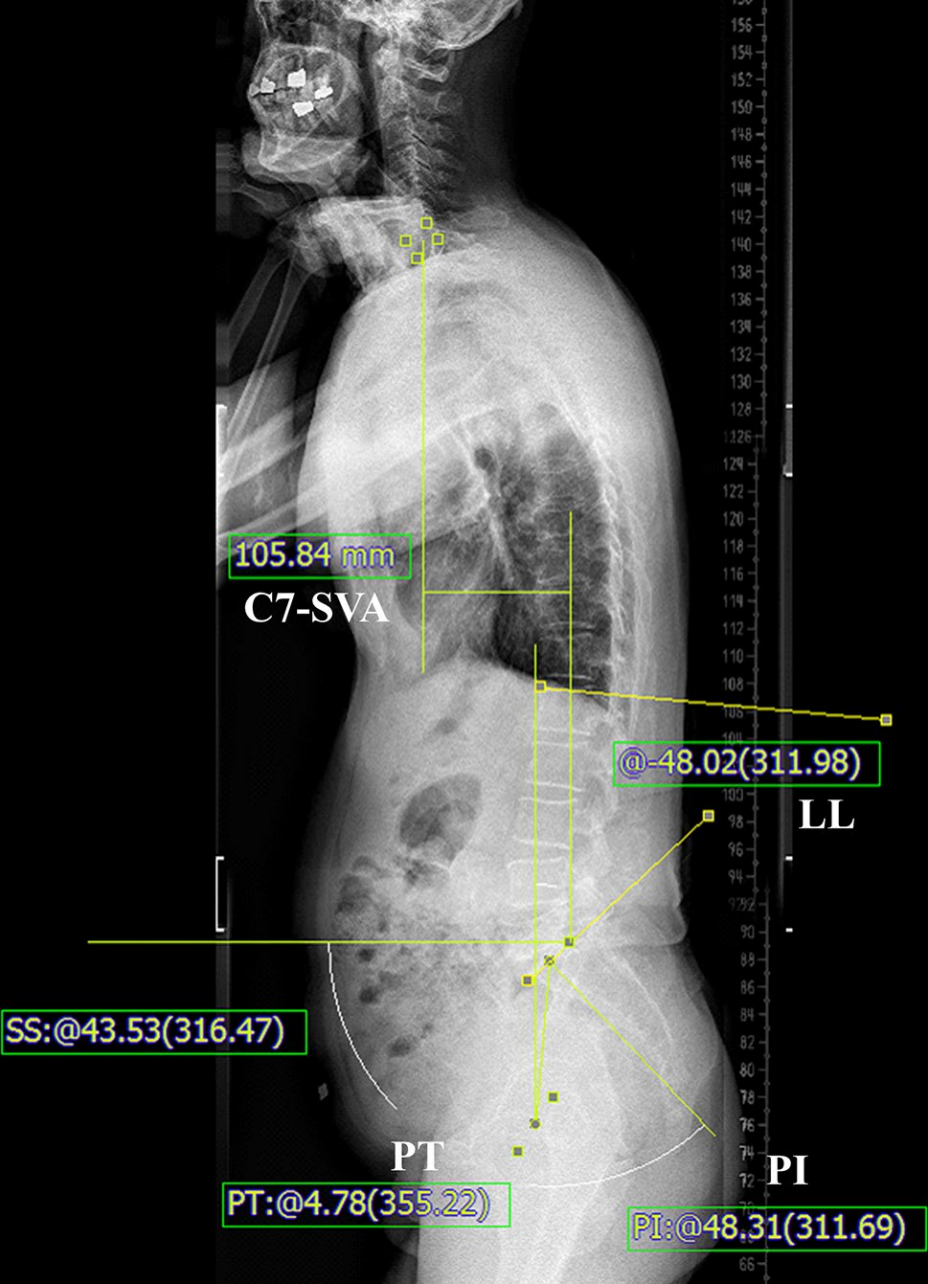
Radiological measurements. The C7 sagittal vertical axis (C7-SVA) is the horizontal distance from the C7 plumb to the posterior-superior corner of S1. Lumbar lordosis (LL) is measured between the superior end plate of L1 and S1. Pelvic incidence (PI) and pelvic tilt (PT) were measured from bicoxo-femora axis (midpoint of centers of the two femoral heads) to the midpoint of superior S1 endplate. The measuring tools are included in the picture archiving and communication systems.

As a routine practice, whole spine radiographs were taken in all patients using 36-inch-long digital lateral radiographic films. Patients were asked to look straight forward, flex the arms approximately 60° to touch the shoulders with each hand and fully extend the hips and knees^4^. Radiological parameters such as C7-SVA, lumbar lordosis (LL), difference between pelvic incidence and lumbar lordosis (PI-LL), and pelvic tilt (PT) were measured by a blinded researcher (Fig. 1). These radiographs were obtained preoperatively, at 3, 6, and 12 months postoperatively, and yearly thereafter. The measurements and the analysis were performed on 150% magnified images using measuring tools in the institution’s picture archiving and communication system (Marosis, version 5483, Infinitt Healthcare, Seoul, Korea), which was run in a Microsoft Windows environment (Microsoft Corp., Redmond, WA, USA)^21^.

### Statistical analysis

The primary end point was the incremental cost-effective ratio (ICER) of fusion surgery and decompression surgery during the first 2 years postoperatively. Quality-adjusted life years (QALYs) were calculated by using the change in the index value of the EQ-5D. Direct costs during hospitalization for the index surgery and outpatient clinics were retrieved from the medical records. Direct costs were defined as the sum of primary costs associated with surgery and secondary costs associated with postoperative course management and unexpected events. Specifically, the primary cost was defined as the sum of the costs associated with the hospital stay (meals, nursing care, laboratory work, medication, physical therapy), radiological examination (computed tomography [CT] and MRI), anesthesia, operation, and surgical equipment (implants, biologics, hemostatic agent, etc.). The secondary cost was defined as the costs associated with regular outpatient clinics, unplanned hospital visits, and readmission. Costs were expressed in terms of 2020 USD, with a 3 percent discount rate applied. Local currencies were exchanged using annual standard exchange rates. The ICER was calculated with the following formula.

$$\begin{aligned} QALYs & ~ = ~\left( {\frac{{preop.~index~\;value~ + ~3month~\;index~\;value}}{2} \times 0.25} \right)~ \\ & \quad + ~\left( {\frac{{3month\;~index\;~value~ + ~6month\;~index~\;value}}{2} \times 0.25} \right) \\ & \quad + ~\left( {\frac{{6month\;~index~\;value~ + ~12month\;~index~\;value}}{2} \times 0.5} \right) \\ ~ & \quad + ~\left( {\frac{{12month\;~index~\;value~ + ~24month\;~index~\;value}}{2} \times 1} \right) \\ \end{aligned}$$

$$QALYs~\;gain = QALYs~{-}~\left( {preoperative~\;index\;~value~ \times 2} \right)$$

$$ICER~ = ~\frac{{\left( {Cost~\;of\;Fusion} \right) - \left( {Cost~\;of\;~Decompression} \right)}}{{\left( {QALYs\;~gain~\;of~\;Fusion} \right) - \left( {QALYs~\;gain\;~of~\;Decompression} \right)}}$$

The baseline characteristics of the D-group and F-group were compared via independent t test or Wilcoxon rank-sum test for continuous variables and a chi-square test or Fisher’s exact test for noncontinuous values.

The clinical outcomes and radiological parameters between groups and times were compared with linear mixed-effects models. The fixed effects included group, time, the interaction between group and time, and factors with *p *values less than 0.2. The random effect was subjects. The least squares means for clinical and radiological parameters were estimated using adjusted 95% confidence intervals (CIs), and comparisons were made between the treatment groups at each time point and between time points within each group.

The minimal clinically important difference (MCID) of the ODI was used to divide patients into improved and nonimproved groups^22,23^. With a multivariate analysis, the factors for clinical improvement were analyzed. The data were analyzed via the SPSS software package version 23.0 (SPSS, Chicago, ILL, USA).

## Results

Seventy-one patients (M:F = 28:43; mean age, 71.39 ± 6.84 years) were included in the present study. Patients were followed-up for a mean of 33.1 ± 9.7 months (24–71). The D group included 40 patients, and the F group included 31 patients. The baseline characteristics of the two groups are shown in Table 1. The D-group had a mean baseline NRS-B score that was 1.41 points higher than that of the F-group (*p* = 0.03). Otherwise, there were no significant differences between the two groups in other baseline clinical characteristics and radiological parameters.

**Table 1:** Baseline characteristics.

	D-group (40)	F-group (31)	*p*-value
**Male/Female**	18/22	10/21	0.398
**Age, yrs ± (SD)**	72.03 ± 6.68	70.58 ± 7.07	0.381
**Height, cm ± (SD)**	159.65 ± 9.65	156.00 ± 7.89	0.084
**Weight, Kg ± (SD)**	65.80 ± 11.57	62.00 ± 8.11	0.109
**BMI, Kg/m**^**2**^** ± (SD)**	25.69 ± 3.01	25.49 ± 2.99	0.791
**F/U, months ± (SD)**	34.43 ± 11.52	31.32 ± 6.35	0.183
**Surgery level**			0.253
1	34	22	
2	6	9	
**SPL grade**			0.398
0	18	10	
1	22	21	
**ASA class**			0.695
I	5	4	
II	34	25	
III	1	2	
**BMD**			0.123
Normal	10	7	
Osteopenia	9	15	
Osteoporosis	6	2	
NA	15	7	
**Preop EQ-5D ± (SD)**	0.412 ± 0.239	0.390 ± 0.216	0.695
**Preop ODI ± (SD)**	24.18 ± 9.14	24.00 ± 8.36	0.933
**Preop NRS-B ± (SD)**	6.00 ± 2.63	7.41 ± 2.34	**0.026**
**Preop NRS-L ± (SD)**	6.63 ± 2.45	6.93 ± 2.02	0.595
**Preop SVA, mm ± (SD)**	80.84 ± 40.94	81.32 ± 46.25	0.963
**Preop LL, ° ± (SD)**	21.21 ± 16.24	24.88 ± 13.32	0.312
**Preop PI-LL, ° ± (SD)**	33.65 ± 14.07	30.69 ± 14.58	0.390
**Preop PT, ° ± (SD)**	26.44 ± 9.56	23.64 ± 10.39	0.243
**Preop PI, ° ± (SD)**	54.75 ± 9.50	55.60 ± 10.09	0.715

*SD* standard deviation, *SPL* spondylolisthesis, *ASA class* american society of anesthesiologists classification, *BMD* bone mineral density, *NA* not applicable, *Preop* preoperative, *EQ-5D* euro-quality of life-5 dimensions, *ODI* oswestry disability index, *NRS-B* numerical rating score of pain on back, *NRS-L* numerical rating score of pain on leg, *SVA* sagittal vertical axis, *LL* lumbar lordosis, *PI-LL* difference between pelvic incidence and lumbar lordosis, *PT* pelvic tilt, *PI* pelvic incidence.

Boldface type indicates statistical significance.

**Figure 2:**
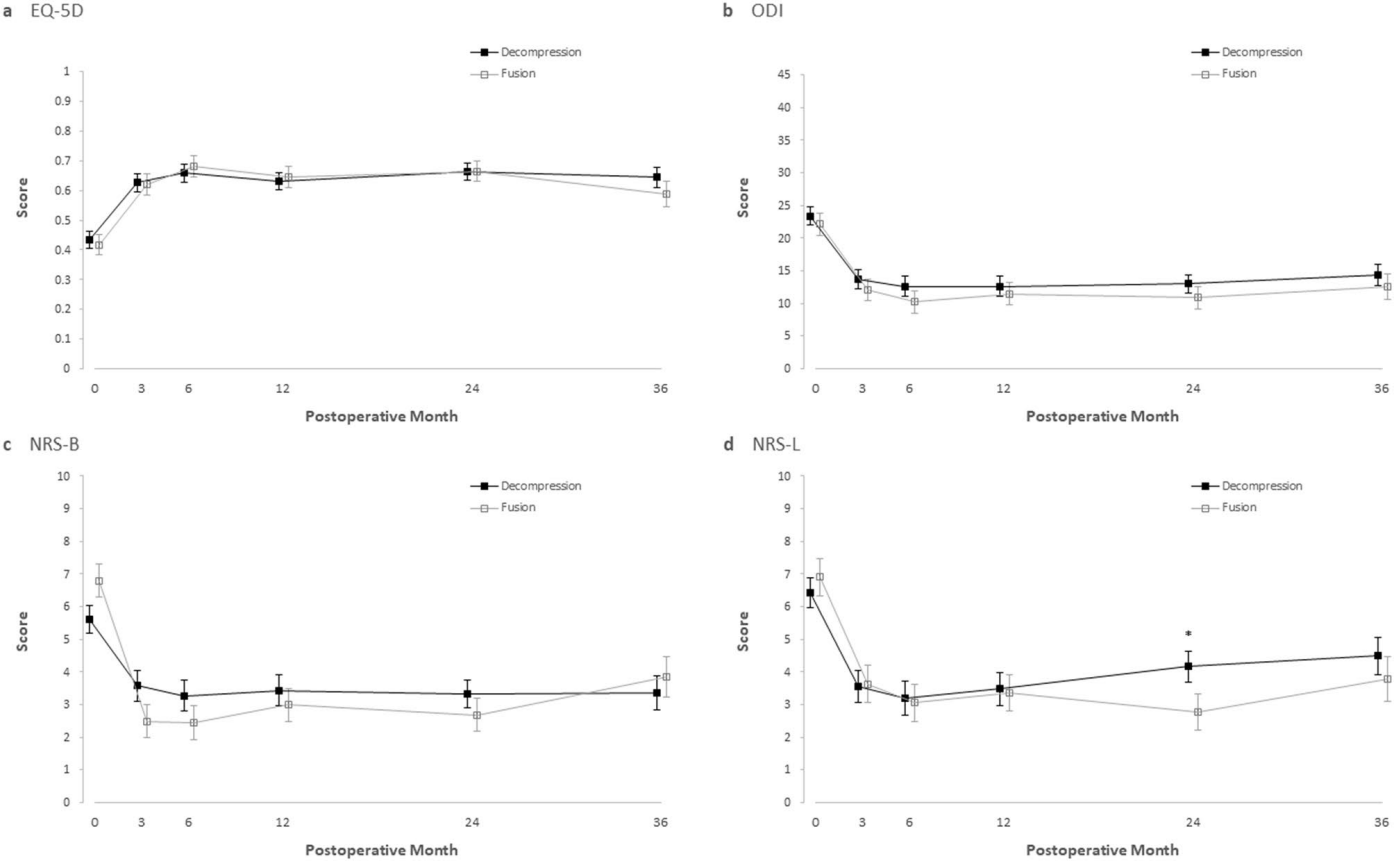
Comparison of clinical outcomes. Shown are adjusted value of Euro-Quality of Life-5 Dimension (EQ-5D; range from 0 to 1, with higher scores indicating better quality of life) (**a**) and Korean version of Oswestry Disability Index (ODI; range from 0 to 45, with higher scores indicating more disability related to back pain) (**b**), numerical rating score of pain on back and leg (NRS-B and NRS-L; range from 0 to 10, with higher scores indicating more pain) (**c** and **d**) before and after surgery, among D-group and F-group. I bars represent standard errors. An asterisk (*) means statistical significance (*p* < 0.05).

**Figure 3:**
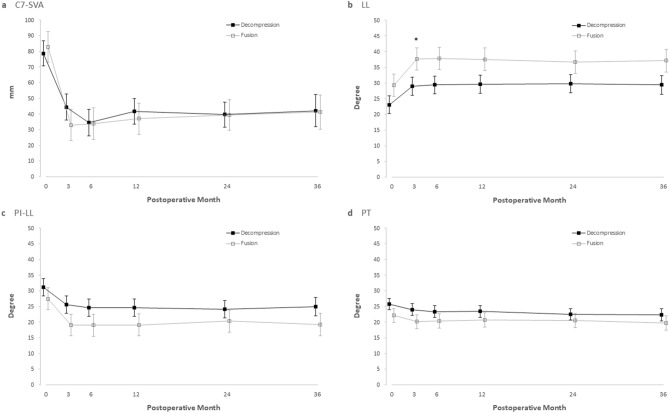
Comparison of radiological parameters. Shown are adjusted value of radiological parameters before and after surgery, among D-group and F-group. I bars represent standard errors. (**a**), C7-Sagittal vertical axis (C7-SVA); (**b**), lumbar lordosis (LL); (**c**), difference between pelvic incidence and lumbar lordosis (PI-LL); (**d**), pelvic tilt (PT). An asterisk (*) means statistical significance (*p* < 0.05).

Figures 2 and 3 show the adjusted values of the clinical and radiological parameters of each group during the follow-up period. Postoperatively, both the D-group and F-group showed clinical improvement across all parameters (*p* < 0.05) without differences between groups (*p* > 0.05) throughout the follow-up period, except for NRS-L (Table 2 and Fig. 2) at two years postoperatively. At 2 years postoperatively, the NRS-L of the D-group was significantly higher in the D-group (4.16; 95% CI, 3.24 to 5.09) than in the F-group (2.76; 95% CI, 1.65 to 3.88) by 1.4 (95% CI, 0.01 to 2.78; *p* = 0.048) (Table 2).

**Table 2:** The adjusted least squares means of clinical and radiological parameters.

Parameter	D-group (40)	F-group (31)	Difference	95% CI	*p*-value
**EQ-5D**					0.836*
Preop	0.436	0.417	− 0.019	− 0.103, 0.066	0.667†
3 mo	0.626	0.620	− 0.006	− 0.093, 0.082	0.894†
6 mo	0.660	0.683	0.023	− 0.067, 0.113	0.613†
1 yr	0.632	0.646	0.014	− 0.072, 0.100	0.744†
2 yr	0.663	0.665	0.002	− 0.083, 0.087	0.958†
3 yr	0.645	0.589	− 0.055	− 0.162, 0.052	0.310†
**ODI (/45)**					0.305*
Preop	23.39	22.13	− 1.26	− 5.35, 2.83	0.544†
3 mo	13.71	12.06	− 1.64	− 5.86, 2.58	0.443†
6 mo	12.62	10.22	− 2.40	− 6.73, 1.93	0.275†
1 yr	12.63	11.46	− 1.16	− 5.38, 3.05	0.586†
2 yr	12.99	10.86	− 2.13	− 6.22, 1.96	0.305†
3 yr	14.39	12.56	− 1.83	− 6.70, 3.03	0.458†
**NRS-B (/10)**					0.619*
Preop	5.62	6.79	1.16	− 0.09, 2.42	0.069†
3 mo	3.57	2.49	− 1.08	− 2.39, 0.23	0.105†
6 mo	3.27	2.45	− 0.82	− 2.18, 0.54	0.237†
1 yr	3.44	2.99	− 0.45	− 1.76, 0.86	0.498†
2 yr	3.33	2.69	− 0.65	− 1.90, 0.61	0.310†
3 yr	3.35	3.84	0.49	− 1.09, 2.08	0.543†
**NRS-L (/10)**					0.564*
Preop	6.42	6.90	0.47	− 0.91, 1.86	0.501†
3 mo	3.55	3.63	0.08	− 1.37, 1.52	0.916†
6 mo	3.19	3.05	− 0.14	− 1.64, 1.35	0.850†
1 yr	3.48	3.36	− 0.12	− 1.56, 1.32	0.870†
2 yr	4.16	2.76	− 1.40	− 2.78, − 0.01	**0.048**†
3 yr	4.49	3.79	− 0.70	− 2.42, 1.02	0.424†
**SVA, mm**					0.829*
Preop	78.74	82.77	4.03	− 19.63, 27.70	0.736†
3 mo	44.43	33.07	− 11.36	− 35.63, 12.90	0.355†
6 mo	34.55	34.00	− 0.55	− 25.14, 24.03	0.965†
1 yr	41.68	37.05	− 4.63	− 28.97, 19.71	0.707†
2 yr	39.67	39.54	− 0.13	− 23.83, 23.56	0.991†
3 yr	42.20	41.35	− 0.85	− 29.53, 27.82	0.953†
**LL, °**					0.070*
Preop	23.06	29.31	6.25	− 2.21, 14.72	0.145†
3 mo	29.00	37.68	8.69	0.19, 17.18	**0.045**†
6 mo	29.37	37.83	8.46	− 0.05, 16.97	0.051†
1 yr	29.64	37.56	7.92	− 0.58, 16.42	0.067†
2 yr	29.79	36.62	6.83	− 1.64, 15.30	0.112†
3 yr	29.40	37.13	7.72	− 1.06, 16.50	0.084†
**PI-LL, °**					0.210*
Preop	31.11	27.44	− 3.67	− 11.95, 4.61	0.379†
3 mo	25.56	19.07	− 6.49	− 14.80, 1.82	0.124†
6 mo	24.58	19.03	− 5.55	− 13.89, 2.78	0.188†
1 yr	24.63	19.09	− 5.54	− 13.87, 2.78	0.188†
2 yr	24.17	20.31	− 3.86	− 12.14, 4.42	0.355†
3 yr	24.93	19.26	− 5.66	− 14.28, 2.96	0.195†
**PT, °**					0.259*
Preop	25.81	22.09	− 3.72	− 9.11, 1.67	0.172†
3 mo	24.02	20.14	− 3.88	− 9.30, 1.54	0.157†
6 mo	23.37	20.36	− 3.01	− 8.44, 2.41	0.271†
1 yr	23.39	20.72	− 2.68	− 8.10, 2.74	0.327†
2 yr	22.48	20.56	− 1.92	− 7.31, 3.47	0.479†
3 yr	22.38	19.71	− 2.68	− 8.34, 2.99	0.350†

*EQ-5D* euro-quality of life-5 dimensions, *ODI* oswestry disability index, *NRS-B* numerical rating score of pain on back, *NRS-L* numerical rating score of pain on leg, *SVA* sagittal vertical axis, *LL* lumbar lordosis, *PI-LL* difference between pelvic incidence and lumbar lordosis, *PT* pelvic tilt, *CI* confidence interval, *Preop* preoperative.

*comparison between groups with time.

^†^post hoc analysis.

Boldface type indicates statistical significance.

Postoperative MRI showed directly or indirectly decompressed lumbar spinal canal (widening of the central canal and disappearance of the nerve root encroachment) in all patients. Radiological parameters also significantly improved in both groups (*p* < 0.05) without a difference between groups (*p* > 0.05) throughout the follow-up period. The adjusted preoperative C7-SVAs were 78.74 mm (95% CI, 62.89 to 94.59) in the D-group and 82.77 mm (95% CI, 63.32 to 102.22) in the F-group (*p* = 0.74). These values were significantly improved to 39.67 mm (95% CI, 23.75 to 55.59, *p* < 0.001) in the D-group and 39.54 mm (95% CI, 20.08 to 58.99, *p* < 0.001) in the F-group at postoperative two years without a difference between groups (*p* = 0.99) (Table 2).

Direct costs by component type are shown in Table 3. Costs during hospitalization for the index surgery were higher in group F (*p* < 0.001), with USD $3,500 in the D-group and USD $8,132 in the F-group. There was no significant difference in costs for postoperative management (*p* = 0.959), with USD $651 in the D-group and USD $669 in F-group. The mean 2-year costs were USD $4,151 in the D-group and USD $8,801 in the F-group (*p* < 0.001).

**Table 3:** Comparison of cost.

	D-group (40)	F-group (31)	*p*-value
	Mean ± SD	Mean ± SD	
**Primary cost ($)**	3499.71 ± 1275.52	8132.27 ± 2007.05	** < 0.001**
Hospital stay	1553.04 ± 1061.53	2057.23 ± 923.24	**0.039**
Length of stay (day)	4.93 ± 1.79	5.55 ± 0.96	0.084
Radiologic exam	517.36 ± 366.58	638.64 ± 506	0.246
Anesthesia	294.68 ± 72.6	513.22 ± 197.13	** < 0.001**
Operation	759.7 ± 165.07	1495.34 ± 282.16	** < 0.001**
Surgical equipment	374.93 ± 118.65	3427.84 ± 833.6	** < 0.001**
**Secondary cost ($)**	650.88 ± 1909.88	668.64 ± 291.92	0.959
Regular visit	274.67 ± 92.93	448.03 ± 204.51	** < 0.001**
Unplanned visit	99.37 ± 274.69	220.62 ± 245.25	0.057
No. of unplanned visits	0.88 ± 1.59	2 ± 1.63	**0.005**
Readmission	276.84 ± 1750.91	0 ± 0	0.383
**Total costs ($)**	4150.59 ± 2399.11	8800.91 ± 2042.63	< 0.001

*SD* standard deviation.

Boldface type indicates statistical significance.

**Figure 4:**
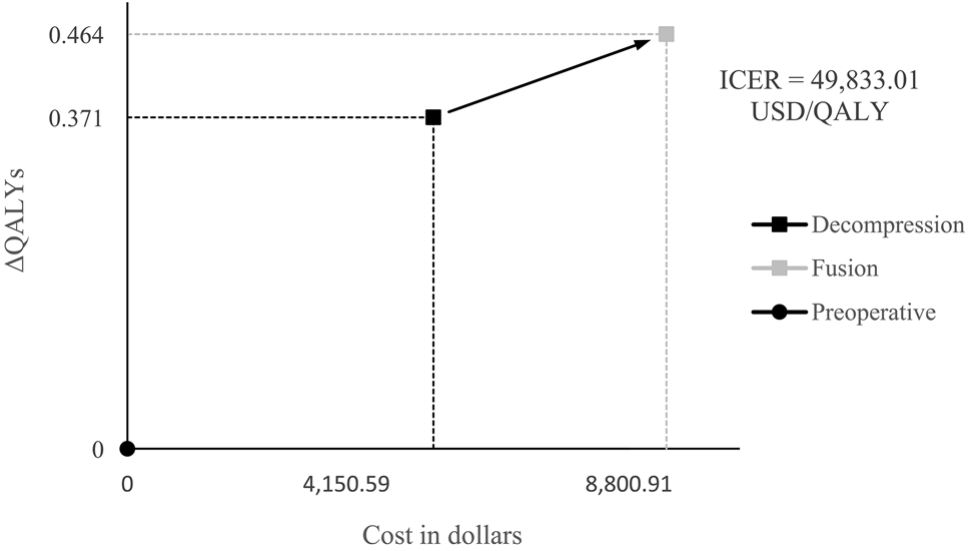
Cost-utility of decompression versus fusion. The costs and QALYs gained by decompression or fusion over a 2-year time horizon.

Figure 4 shows the costs and QALYs gained in the 2 groups. The mean QALYs gained were 0.371 in the D-group and 0.464 in the F-group during the 2-year follow-up period. The difference in QALYs gained between the two groups was not statistically significant (*p* = 0.33). The ICER of the F-group over the D-group was $49,833 USD/QALY (*p* < 0.001).

Clinically, 60/71 (84.5%) patients showed improved ODI, and 45/71 (67.2%) patients improved more than the MCID of ODI. The most significant predictive factor(s) was high preoperative NRS-B (*p* < 0.01), while the method of surgery or radiological parameters did not significantly influence the outcomes (*p* > 0.05) (Table 4). There were no cases of surgery-related complications, such as dura tears or surgical site infections. However, 3 of the 40 patients who underwent decompression eventually underwent fusion at 18, 30, and 31 months after surgery. When calculating the ICER at two years, the additional cost of reoperation was considered for one patient who underwent additional fusion at postoperative 18 months.

**Table 4:** Risk factors for the non-improvement of ODI* at postoperative 2 years.

	Univariate			Multivariate		
	*p*-value	OR	95% CI	*p*-value	OR	95% CI
Fusion	0.802	1.14	0.41, 3.18			
Female	0.436	0.66	0.23, 1.87	0.073	14.23	0.78, 259.89
Age, yrs	0.759	0.99	0.93, 1.05			
Height, cm	0.299	1.03	0.97, 1.09	0.114	1.12	0.97, 1.28
Weight, Kg	0.746	1.01	0.96, 1.06			
BMI, Kg/m^2^	0.595	0.96	0.81, 1.13			
2 level surgery	0.131	2.53	0.76, 8.46	0.081	3.73	0.85, 16.34
SPL	0.805	0.88	0.31, 2.49			
Preop NRS-B	**0.011**	0.75	0.61, 0.94	**0.006**	0.68	0.52, 0.89
Preop NRS-L	0.264	0.88	0.70, 1.10			
Preop SVA, mm	0.377	0.99	0.98, 1.01			
Preop LL, °	0.922	1.00	0.97, 1.03			
Preop PI-LL, °	0.156	0.97	0.94, 1.01			
Preop PT, °	0.165	0.96	0.91, 1.02			
Preop PI, °	0.095	0.95	0.90, 1.01	0.078	0.94	0.87, 1.01

*SPL* spondylolisthesis, *Preop* preoperative, *NRS-B* numerical rating score of pain on back, *NRS-L* numerical rating score of pain on leg, *SVA* sagittal vertical axis, *LL* lumbar lordosis, *PI-LL* difference between pelvic incidence and lumbar lordosis, *PT* pelvic tilt, *PI* pelvic incidence, *OR* odds ratio, *CI* confidence interval.

Nagelkerke R^2^ statistic = 0.284 and Hosmer and Lemeshow goodness of fit test was not significant at 5% (*P* = 0.792).

*The minimal clinically important difference of Oswestry disability index was set as 8/50^21,22^.

Boldface type indicates statistical significance.

## Discussion

### Overview of the results

The objective of this study was to analyze the cost utility comparing lumbar decompression surgery and lumbar fusion surgery for elderly patients with LSS and sagittal imbalance. This study showed that the clinical and radiological outcomes of lumbar decompression surgery were not significantly different from those of fusion surgery, albeit with less improvement in leg pain at 2 years. In addition, decompression surgery saved $49,833 US dollars/QALY compared to fusion surgery.

Lumbar spinal stenosis and surgical options for elderly patients.

Recently, the incidence of spinal disease has increased, as has the surgical volume^3,24^. As many societies continue to age, the number of degenerative spinal diseases is also increasing^1,24^. Among lumbar degenerative diseases, spinal stenosis is the most common cause of surgery and incurs a huge economic burden^2,3,25^. The surgical options for lumbar spinal stenosis are largely divided into decompression only and decompression and fusion surgery^5^. Recently, the proportion of fusion surgery has grown much more than that of decompression surgery^25–28^. It has been reported that fusion surgery is more advantageous than decompression only surgery when there is instability or accompanied by diseases such as spondylolisthesis or deformity^6,29–31^. Nonetheless, the role of instrumented fusion surgery for elderly patients with stable spinal stenosis and sagittal imbalance is not straightforward^32,33^. Several studies found that the impact of surgeon and hospital factors was more significant than the patient factor in determining the surgical method^34,35^. In particular, no consensus has been reached on selecting a surgical method for elderly patients with stable LSS and sagittal imbalance^5,33^. Although postoperative sagittal imbalance has been correlated with less satisfactory outcomes and mechanical failure, completely restoring the “ideal” sagittal balance of younger populations may be too strict for elderly patients^28,36–39^. Recent studies have shown that sagittal balance measured by C7-SVA can in some cases be restored by decompression-only surgery, although the restoration may not be as complete as with fusion surgery^4,5,14^. A recent randomized controlled trial also supported decompression surgery over instrumented fusion surgery for LSS without instability^6,12,13^. In these regards, symptomatic improvement without complete radiological correction may be a reasonable option for certain elderly patients considering the added cost and morbidity of fusion surgery and similar outcomes achieved^5,15,16^. However, there is concern that it is unclear whether the sagittal imbalance is one of a symptom of LSS or a presentation of spinal deformity. Those were not clearly differentiated with radiological parameters or clinical symptom, since those factors were often overlap^40^. In this study, we included patients with severe lumbar spinal stenosis and the main symptom of neurogenic claudication or pain. Once their main symptom was relived after surgery, the residual deformity was tolerable to the patients because deformity was not a main problem before surgery. The present study showed that clinical and radiological outcomes were similar between decompression and fusion surgery. Although leg pain scores were better in the F versus D group at 2 years postoperatively, they still remained improved versus the preoperative scores, and they did not increase by 1.6, which is the minimal clinical importance difference (MCID) of leg pain^41^. In addition, this difference at 2 years did not influence functional status assessed by ODI and EQ-5D. Therefore, the clinical outcomes of decompression surgery seemed to be as acceptable as those of fusion surgery. Yavin et al. reported that fusion surgery provided slightly greater relief of pain than decompression surgery, but disability and patient satisfaction were similar and suggested a limited role of fusion in the management of spinal stenosis through systematic review^42^. Although the increase in leg pain was less than the MCID, leg pain at 2 years in this study was noteworthy. Because MRI or CT scans were not taken, the cause of increased leg pain is not clear, but ongoing degeneration may cause recurrent stenosis after decompression surgery but not after fusion surgery. The long-term consequence of the ongoing degenerative process and its influence on functional status requires further follow-up.

Interestingly, radiological parameters were not different between the two groups. Sagittal balance, measured by C7-SVA, was significantly improved in both groups without significant difference between groups. Patients with LSS tend to lean forward as a compensatory mechanism to enlarge the spinal canal^5^. This is different from a deformity. Therefore, removal of the LF with decompression surgery may allow the patient to stand upright without a compensatory forward leaning posture. Improvements in pain and function after surgery may also facilitate upright posture^4^. These may be reasons for the restoration of sagittal balance. However, despite similar radiological outcomes between OLIF and conventional posterior decompression and fusion surgery, the restoration of sagittal balance by indirect decompression of OLIF may undermine the effect of fusion surgery^43^. In this study, successful indirect decompression, the widening of the central canal and the disappearance of the nerve root encroachment, was confirmed with postoperative MRI. Nonetheless, this is an issue to be studied in future studies. In addition, the patients in this study had a modestly positive sagittal imbalance, with an average C7-SVA of approximately 8 cm. The results of this study may not apply equally to patients with severe sagittal imbalance or those with a true sagittal plane deformity^5^.

Many reports demonstrate that lumbar decompression without fusion is more cost-effective than fusion in LSS^11,44^. This study showed a similar result in elderly patients with LSS and sagittal imbalance, and decompression surgery could be a surgical option for them. Despite the current results and previous studies, decompression surgery alone was not recommended for patients with severe sagittal imbalance (SVA > 9.5 cm)^10^ because a significant sagittal imbalance would persist after decompression alone^5^. A recent systematic review showed that LSS, if associated with preoperative PI-LL less than 20 degrees and C7-SVA less than 8 cm, may not require additional corrective fusion procedure^5^. These issues should be considered in a shared decision-making process.

In addition to clinical and radiological outcomes, medical cost is also an important issue. In all countries, fusion surgery is more costly than decompression surgery and places a large burden on health insurance^3,7,11,24,25,29,44^. All patients in this study were beneficiaries of the national health insurance system, and the results showed that fusion surgery cost $49,833 US dollars/QALY more than decompression surgery. And most of this difference was due to costs associated with surgical equipment, surgery, and anesthesia. In this study, expenses at other hospitals and indirect costs, such as job loss and unpaid caregiver's time, were not considered. Considering that the time to return to job after fusion is usually longer than that of decompression, the ICER of fusion over decompression may be higher when indirect medical cost is included^45^. However, QALYs gained after OLIF may underestimate QALYs gained after fusion surgery because decompression was indirect with OLIF and the effect of decompression may be less than that of posterior fusion surgery^46,47^. Although the clinical outcomes of indirect decompression are not inferior to those of direct decompression^43^, it cannot be excluded that OLIF might have a suboptimal QALY gain compared to that of direct posterior decompression and fusion surgery.

### Limitations

First, this study was not a randomized controlled trial (RCT) and had a chance of selection bias. Patients who underwent fusion surgery had more severe back pain than patients who underwent decompression surgery, and this finding implied that the selection of the surgical method was not randomized. However, the surgical method was decided during a shared decision-making process, and it was decided by both patients and surgeons considering clinical symptoms and radiological findings. Although there was chance of selection bias, this study showed the results of an actual clinical situation, respecting the decision of patients. Second, the number of patients was small, which could lead to both type I and II errors. In addition, the difference in leg pain scores at 2 years postoperatively implies that longer follow-up is necessary. Further studies with a large number of patients and longer follow-up periods are required to verify the current results. Third, the direct medical cost in the Republic of Korea is relatively lower than that in other countries, and the ICER may have been underestimated^24–26^. In particular, it was found that the cost differences of surgical equipment, operation, and anesthesia were quite large, so ICER would be higher in other countries where these costs are expensive. Fourth, despite similar clinical and radiological outcomes of OLIF with those of conventional fusion surgery, indirect decompression of OLIF may undermine the effect of restoring sagittal balance and QALY gain versus posterior direct decompression and fusion surgery. Fifth, lifestyle, occupational activities, and strength of back muscles may have influenced the outcomes, but those factors were not controlled in the analysis^38^. Finally, it is difficult to generalize the results of this study to patients in other countries because of the sedentary lifestyle, national health insurance system, low direct hospital cost of the current study, and various willingness-to-pay costs in different countries. Nevertheless, the present study showed that nonfusion surgery may be a viable option for certain elderly patients with LSS, stable segments with up to grade 1 spondylolisthesis, and modest sagittal imbalance (up to 8 cm C7-SVA). This result may be considerable in a shared decision-making process with the patient and lead to a reduction in the overall burden of healthcare.

## Conclusions

For elderly patients with LSS, stable segments with up to a grade 1 spondylolisthesis, and modest sagittal imbalance (up to 8 cm C7-SVA), both decompression and fusion surgery showed similar clinical and radiological outcomes. In addition, fusion surgery cost $49,833 US dollars/QALY compared to decompression surgery. Decompression-only surgery may be a viable surgical option for certain elderly patients in the shared decision-making process.

## References

[1] Fehlings MG (2015). The aging of the global population: The changing epidemiology of disease and spinal disorders. Neurosurgery.

[2] Kim CH (2013). Reoperation rate after surgery for lumbar spinal stenosis without spondylolisthesis: a nationwide cohort study. Spine J..

[3] Ziino C, Mertz K, Hu S, Kamal R (2020). Decompression with or without fusion for lumbar stenosis: A cost minimization analysis. Spine (Phila Pa 1976).

[4] Shin EK (2017). Sagittal imbalance in patients with lumbar spinal stenosis and outcomes after simple decompression surgery. Spine J..

[5] Ogura Y, Kobayashi Y, Shinozaki Y, Ogawa J (2020). Spontaneous correction of sagittal spinopelvic malalignment after decompression surgery without corrective fusion procedure for lumbar spinal stenosis and its impact on clinical outcomes: A systematic review. J. Orthop. Sci..

[6] Ghogawala Z (2016). Laminectomy plus fusion versus laminectomy alone for lumbar spondylolisthesis. N. Engl. J. Med..

[7] Weinstein JN, Lurie JD, Olson PR, Bronner KK, Fisher ES (2006). United States' trends and regional variations in lumbar spine surgery: 1992–2003. Spine (Phila Pa 1976).

[8] Irwin ZN (2005). Variation in surgical decision making for degenerative spinal disorders. Part I: Lumbar spine. Spine (Phila Pa 1976).

[9] Bae JS, Jang JS, Lee SH, Kim JU (2012). A comparison study on the change in lumbar lordosis when standing, sitting on a chair, and sitting on the floor in normal individuals. J Korean Neurosurg Soc.

[10] Lafage R (2016). Defining spino-pelvic alignment thresholds: Should operative goals in adult spinal deformity surgery account for age?. Spine (Phila Pa 1976).

[11] Devin CJ (2015). A cost-utility analysis of lumbar decompression with and without fusion for degenerative spine disease in the elderly. Neurosurgery.

[12] Forsth P (2016). A randomized, controlled trial of fusion surgery for lumbar spinal stenosis. N. Engl. J. Med..

[13] Austevoll IM (2021). Decompression with or without fusion in degenerative lumbar spondylolisthesis. N. Engl. J. Med..

[14] Ogawa R (2015). Total en bloc spondylectomy for locally aggressive vertebral hemangioma causing neurological deficits. Case Rep. Orthop..

[15] Ogura Y (2019). Impact of sagittal spinopelvic alignment on clinical outcomes and health-related quality of life after decompression surgery without fusion for lumbar spinal stenosis. J. Neurosurg. Spine.

[16] Hikata T (2015). Impact of sagittal spinopelvic alignment on clinical outcomes after decompression surgery for lumbar spinal canal stenosis without coronal imbalance. J. Neurosurg. Spine.

[17] Yoshida Y (2022). Association between paravertebral muscle mass and improvement in sagittal imbalance after decompression surgery of lumbar spinal stenosis. Spine (Phila Pa 1976).

[18] Wong AYL, Karppinen J, Samartzis D (2017). Low back pain in older adults: Risk factors, management options and future directions. Scoliosis Spinal Disord..

[19] Kim DY (2005). Validation of the Korean version of the oswestry disability index. Spine (Phila Pa 1976).

[20] Brooks R (1996). EuroQol: The current state of play. Health Policy.

[21] Kim CH (2012). Validation of a simple computerized tool for measuring spinal and pelvic parameters. J. Neurosurg. Spine.

[22] Parker SL (2012). Minimum clinically important difference in pain, disability, and quality of life after neural decompression and fusion for same-level recurrent lumbar stenosis: Understanding clinical versus statistical significance. J. Neurosurg. Spine.

[23] Comins J (2020). Psychometric validation of the danish version of the oswestry disability index in patients with chronic low back pain. Spine (Phila Pa 1976).

[24] Lee CH, Chung CK, Kim CH, Kwon JW (2018). Health care burden of spinal diseases in the Republic of Korea: Analysis of a nationwide database from 2012 through 2016. Neurospine.

[25] Kim CH (2018). Increased volume of surgery for lumbar spinal stenosis and changes in surgical methods and outcomes: A nationwide cohort study with a 5-year follow-up. World Neurosurg..

[26] Kim CH (2019). Increased proportion of fusion surgery for degenerative lumbar spondylolisthesis and changes in reoperation rate: A nationwide cohort study with a minimum 5-year follow-up. Spine (Phila Pa 1976).

[27] Deyo RA, Mirza SK, Martin BI (2011). Error in trends, major medical complications, and charges associated with surgery for lumbar spinal stenosis in older adults. JAMA.

[28] Kim CW, Hyun SJ, Kim KJ (2020). Surgical impact on global sagittal alignment and health-related quality of life following cervical kyphosis correction surgery: Systematic review. Neurospine.

[29] Tosteson AN (2011). Comparative effectiveness evidence from the spine patient outcomes research trial: Surgical versus nonoperative care for spinal stenosis, degenerative spondylolisthesis, and intervertebral disc herniation. Spine (Phila Pa 1976).

[30] Resnick DK (2014). Guideline update for the performance of fusion procedures for degenerative disease of the lumbar spine. Part 10: Lumbar fusion for stenosis without spondylolisthesis. J. Neurosurg. Spine.

[31] Peul WC, Moojen WA (2016). Fusion for lumbar spinal stenosis-safeguard or superfluous surgical implant?. N. Engl. J. Med..

[32] Aebi M (2005). The adult scoliosis. Eur. Spine J..

[33] Hori Y (2019). Does sagittal imbalance impact the surgical outcomes of short-segment fusion for lumbar spinal stenosis associated with degenerative lumbar scoliosis?. J. Orthop. Sci..

[34] Lubelski D (2018). Variability in surgical treatment of spondylolisthesis among spine surgeons. World Neurosurg..

[35] Huang M (2021). Impact of surgeon and hospital factors on surgical decision-making for grade 1 degenerative lumbar spondylolisthesis: A quality outcomes database analysis. J. Neurosurg. Spine.

[36] Korovessis P, Repantis T, Papazisis Z, Iliopoulos P (2010). Effect of sagittal spinal balance, levels of posterior instrumentation, and length of follow-up on low back pain in patients undergoing posterior decompression and instrumented fusion for degenerative lumbar spine disease: A multifactorial analysis. Spine (Phila Pa 1976).

[37] Jang JS (2009). Can patients with sagittally well-compensated lumbar degenerative kyphosis benefit from surgical treatment for intractable back pain?. Neurosurgery.

[38] Kim KH (2021). The important role of paraspinal muscle quality for maintaining sagittal balance while walking: Commentary on "Correlation of paraspinal muscle mass with decompensation of sagittal adult spinal deformity after setting of fatigue post 10-minute walk". Neurospine.

[39] Montenegro TS (2021). Are lumbar fusion guidelines followed? A survey of North American spine surgeons. Neurospine.

[40] Pourtaheri S (2017). Pelvic retroversion: A compensatory mechanism for lumbar stenosis. J. Neurosurg. Spine.

[41] Copay AG (2008). Minimum clinically important difference in lumbar spine surgery patients: a choice of methods using the oswestry disability Index, medical outcomes study questionnaire short form 36, and pain scales. Spine J..

[42] Yavin D (2017). Lumbar fusion for degenerative disease: A systematic review and meta-analysis. Neurosurgery.

[43] Zhu HF (2021). Comparison of oblique lateral interbody fusion (OLIF) and minimally invasive transforaminal lumbar interbody fusion (MI-TLIF) for treatment of lumbar degeneration disease: A prospective cohort study. Spine (Phila Pa 1976).

[44] Aichmair A (2017). Cost-effectiveness of conservative versus surgical treatment strategies of lumbar spinal stenosis in the Swiss setting: Analysis of the prospective multicenter lumbar stenosis outcome study (LSOS). Eur. Spine J..

[45] Singh, S. *et al.* Time to return to work after elective lumbar spine surgery. *J. Neurosurg. Spine* 1–9. 10.3171/2021.2.SPINE202051 (2021). Online ahead of print. 10.3171/2021.2.SPINE202051 34560636

[46] Souslian, F. G. & Patel, P. D. Review and analysis of modern lumbar spinal fusion techniques. *Br. J. Neurosurg.* 1–7. 10.1080/02688697.2021.1881041 (2021). Online ahead of print. 10.1080/02688697.2021.1881041 34263676

[47] Shimizu T, Fujibayashi S, Otsuki B, Murata K, Matsuda S (2021). Indirect decompression via oblique lateral interbody fusion for severe degenerative lumbar spinal stenosis: A comparative study with direct decompression transforaminal/posterior lumbar interbody fusion. Spine J..

